# The NMR signature of maltose-based glycation in full-length proteins

**DOI:** 10.1007/s10858-023-00432-5

**Published:** 2023-12-20

**Authors:** Pauline Defant, Christof Regl, Christian G. Huber, Mario Schubert

**Affiliations:** 1 Department of Biosciences and Medical Biology, University of Salzburg, Hellbrunnerstrasse 34, 5020 Salzburg, Austria; 2 Department of Biology, Chemistry and Pharmacy, Freie Universität Berlin, Takustr. 3, 14195 Berlin, Germany

**Keywords:** Glycation, NMR spectroscopy, Biotherapeutics, Posttranslational modifications, Amadori product, Maltose

## Abstract

Reducing sugars can spontaneously react with free amines in protein side chains leading to posttranslational modifications (PTMs) called glycation. In contrast to glycosylation, glycation is a non-enzymatic modification with consequences on the overall charge, solubility, aggregation susceptibility and functionality of a protein. Glycation is a critical quality attribute of therapeutic monoclonal antibodies. In addition to glucose, also disaccharides like maltose can form glycation products. We present here a detailed NMR analysis of the Amadori product formed between proteins and maltose. For better comparison, data collection was done under denaturing conditions using 7 M urea-d_4_ in D_2_O. The here presented correlation patterns serve as a signature and can be used to identify maltose-based glycation in any protein that can be denatured. In addition to the model protein BSA, which can be readily glycated, we present data of the biotherapeutic abatacept containing maltose in its formulation buffer. With this contribution, we demonstrate that NMR spectroscopy is an independent method for detecting maltose-based glycation, that is suited for cross-validation with other methods.

**Supplementary Information:**

The online version contains supplementary material available at 10.1007/s10858-023-00432-5.

## Introduction

**Fig. 1:**
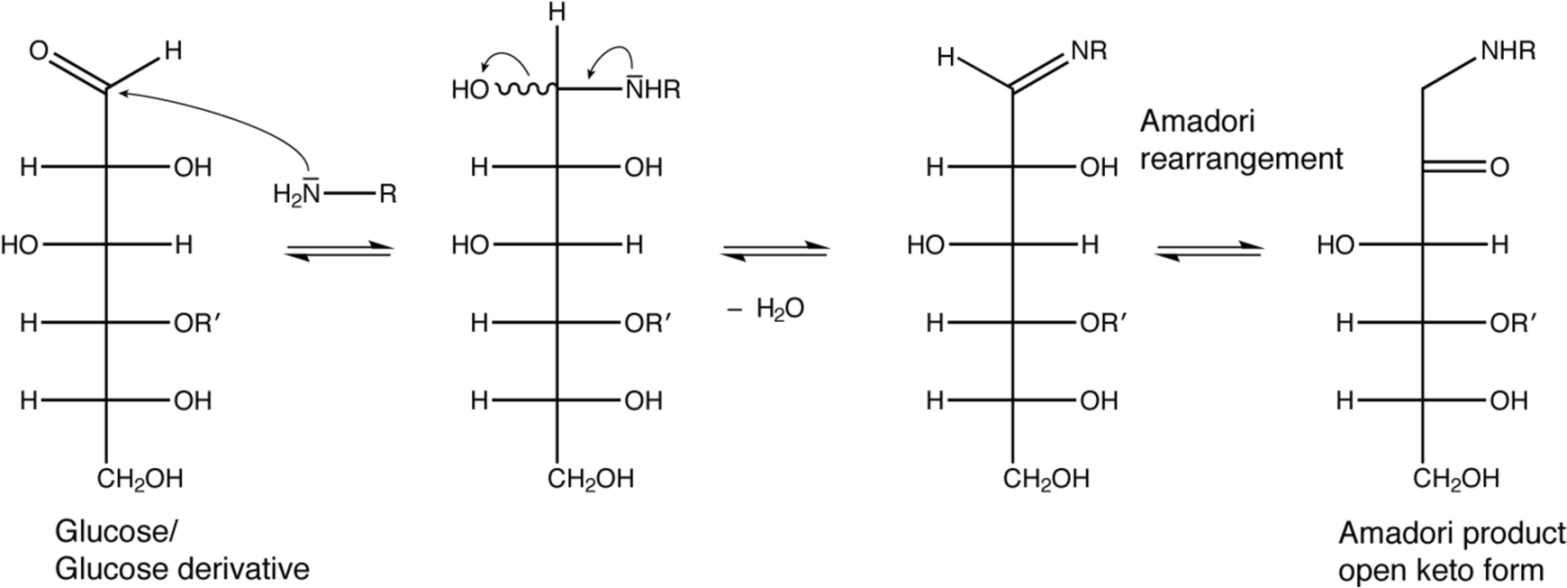
Glycation mechanism between a reductive saccharide (here glucose derivative) and a primary amine. In the case of maltose, R′ stands for α-d-glucose, in case of glucose R′ is a hydrogen. After Amadori rearrangement a derivative of d-fructose is formed

**Fig. 2:**
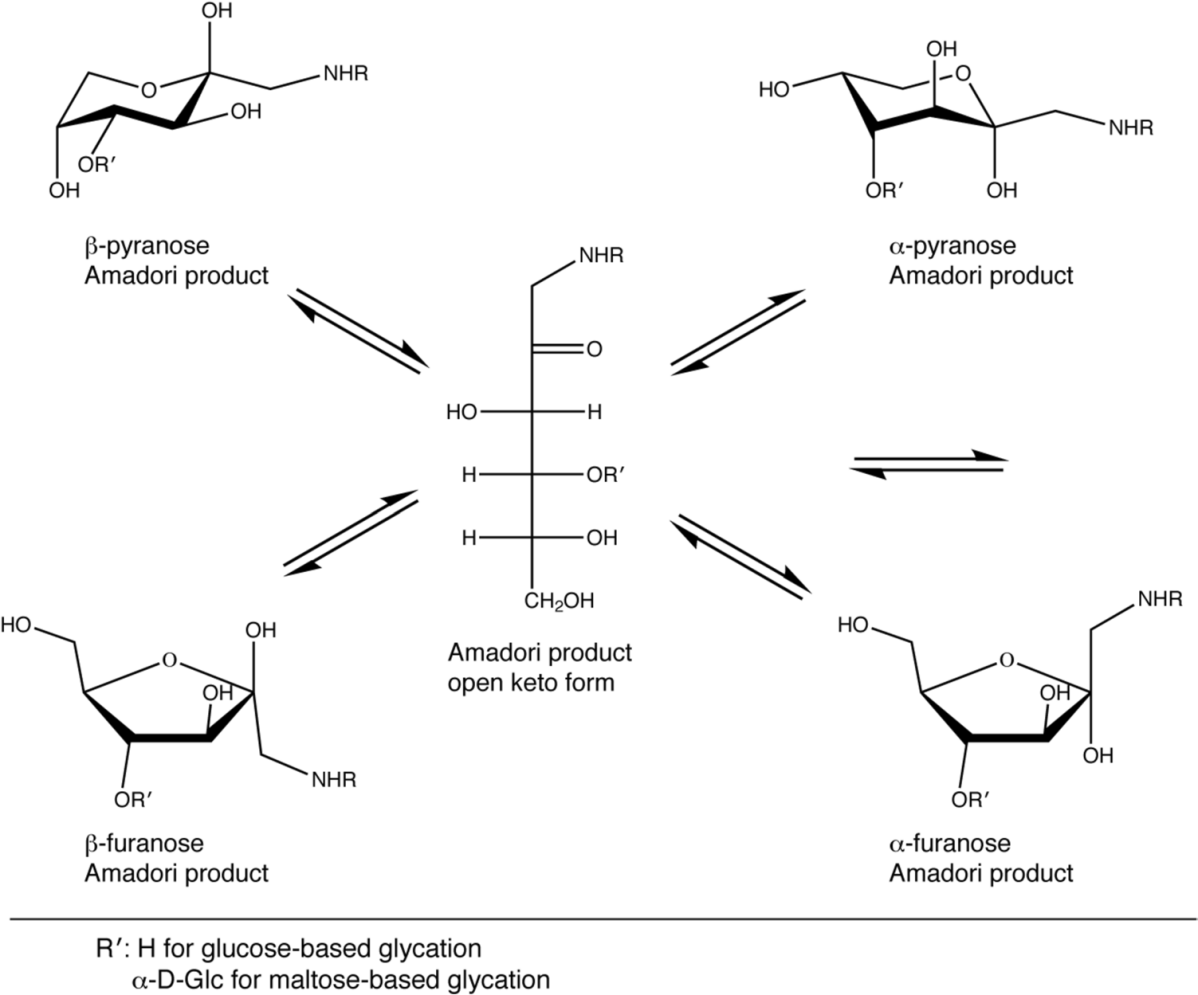
The expected equilibrium of the different forms of maltose-based glycation products in analogy to glucose-based glycation

Carbohydrates with a free reducing end can react spontaneously with amines of proteins. This process is called glycation and stands in sharp contrast to glycosylation, which comprises all enzymatic attachments of mono- or oligosaccharides to amino acid side chains in proteins. Spontaneous glycation between an aldehyde group of an aldose moiety leads via a Schiff base and the Amadori rearrangement to Amadori products (Fig. 1), which typically exist as a mixture of different forms (Fig. 2). For example, in the case of glucose-based glycation the Amadori product exists as a mixture of 70% β-pyranose, 13% α-furanose, 13% β-furanose and 4% α-pyranose form (Mossine et al. 1994; Kaufmann et al. 2016; Moises et al. 2022). Glycation is a reversible process (Xu et al. 2022) unless the Amadori products react further to advanced glycation end products (AGEs) (Goldin et al. 2006).

Glycation of proteins with glucose is very abundant. It occurs for example in the bloodstream, in which always a certain glucose concentration is present (Xu et al. 2022), or during the production of therapeutic proteins due to glucose in the medium and cytosol (Quan et al. 2008; Beck and Liu 2019). Glucose-based glycation is very well studied, also because it is a critical quality attribute in therapeutic proteins (Alt et al. 2016; Sjögren et al. 2016). It is mainly detected and monitored by mass-spectrometry coupled with high-performance liquid chromatography (Schmutzler and Hoffmann 2022; Rabbani et al. 2016; Soboleva et al. 2017; Zhang et al. 2011) but also recently by NMR spectroscopy (Kaufmann et al. 2016; Moises et al. 2022).

Glycation with maltose is much less investigated (Leblanc et al. 2016; Montgomery et al. 2010; Krause et al. 2003), although it is also highly relevant for protein therapeutics, as high maltose concentrations are used in drug formulation buffers of e.g. abatacept and tositumomab (Strickley and Lambert 2021; Lynaugh et al. 2013).

After observing unknown NMR correlation patterns in a sample of abatacept, an Fc fusion protein with maltose in the formulation buffer, we suspected glycation by maltose as origin. Indeed, maltose-based glycation in abatacept was previously detected by HPLC-MS (Lynaugh et al. 2013). To find potential characteristic NMR correlation patterns, we undertook a systematic NMR analysis of Amadori products formed from maltose and the model protein BSA.

2D NMR spectroscopy developed into a versatile technique for the identification of different PTMs. For example glycans in small or even large proteins can be directly observed with ^1^H-^13^C correlations due to fast tumbling or their intrinsic flexibility (Unione et al. 2019; de Beer et al. 1994). However, to ensure that the signals of the PTMs are not impaired by line broadening especially in large proteins an approach for denaturing was developed that is compatible with ^1^H-^13^C correlations (Schubert et al. 2015). Using deuterated urea in D_2_O, in which the lyophilized protein of interest is dissolved, and adding reducing agent for eliminating disulfide bonds results in simplified spectra showing signals with random coil chemical shifts. Ideally, each PTM is recognized by a unique chemical shift correlation pattern that is sufficiently different from random coil correlations of the proteinogenic amino acids within a protein as illustrated for such diverse modifications as specific glyco epitopes (Unione et al. 2019; Schubert et al. 2015; Hinterholzer et al. 2022; Hargett et al. 2021; Peng et al. 2018), oxidation products (Hinterholzer et al. 2020), deamidation products (Grassi et al. 2017), pyroglutamate (Hinterholzer et al. 2019), aspartate isomerization (Hinterholzer et al. 2021) and glycation (Moises et al. 2022). Typically the observed patterns are so characteristic that an unambiguous identification of a certain modification is achieved, which is very reliable and orthogonal to HPLC-MS^2^ techniques.

Here we present characteristic NMR correlation patterns of maltose-based glycation in ^1^H-^13^C and ^1^H-^1^H correlation spectra. These patterns are suitable for an unambiguous detection of glycation by maltose in proteins. Independently we use HPLC-MS/MS to confirm maltose glycation. We illustrate that the presented NMR approach is complementary to MS/MS-based methods and is suited as an independent standard for cross-validation.

## Material and methods

### Procedure for the glycation of bovine serum albumin with maltose

Bovine serum albumin (BSA, Sigma A7030, 70 mg) was dissolved in 3.3 mL of 50 mM KH_2_PO_4_/K_2_HPO_4_ pH 7.4, 100 mM NaCl buffer and mixed with 6.7 mL 276.4 mM maltose monohydrate (Fluka 63419). The solution was incubated for 11 days at 40 °C. After incubation the buffer was exchanged by ultrafiltration to ddH_2_O using Amicon (Sigma Aldrich Amicon Ultra-15, UFC9030) with a cutoff of 30 kDa. 100 µL of the obtained 1.5 mL solution was taken for MS/MS analysis. Subsequently, the remaining sample was lyophilized and then dissolved in 550 µL of a 7 M urea-d_4_ (Armar Chemicals 049500,3041) solution in D_2_O (Armar Chemicals 014400,0010) for NMR analysis. The pH* (uncorrected readout measured in D_2_O) was adjusted to 7.4 by adding 3% DCl in D_2_O (Armar Chemicals 042100.0035). To reduce the disulfide bonds DTT-d_10_ (Cambridge Isotope Laboratories DLM-2622-0) was added to a concentration of 67 mM. The sample was heated to 60 °C for 15 min. The final protein concentration was ~ 1.9 mM.

### Sample preparation of abatacept for NMR analysis

The sample preparation was previously described (Hinterholzer et al. 2022). In brief, abatacept (ORENCIA®, Bristol Myers Squibb; Lot. OE61132, exp. 08/2012) 60 mg in 2.4 mL formulation buffer was dialyzed twice against 4 L ddH_2_O overnight using a SpectraPor membrane with a cut-off of 3.5 kDa. After lyophilization the sample was dissolved in 650 μL of a 7 M urea-d_4_ (98 atom%D, ARMAR Chemicals) solution in D_2_O. DTT-d_10_ (Cambridge Isotope Laboratories) was added to a concentration of 15 mM, and after an incubation for 15 min at 60 °C, the pH* was adjusted to 7.4 using NaOD (Armar Chemicals).

### NMR spectroscopy

Spectra were measured on a 600 MHz Bruker Avance III HD spectrometer equipped with a ^1^H/^13^C/^15^N/^31^P quadruple-resonance room temperature probe at 298 K. All samples were measured in a standard 5 mm NMR tube (Armar, Type 5TA) with a volume of 500 µL or 550 µL. For assigning the resonances of the Amadori products, the following 2D experiments were recorded: ^1^H–^13^C HSQC, ^1^H–^13^C HMBC (hmbcgpndqf), ^1^H–^1^H TOCSY with mixing times of 100 ms and 12 ms, ^1^H–^1^H COSY (cosygpppqf), ^1^H–^13^C HSQC–TOCSY (hsqcdietgpsisp.2) with mixing times of 13 ms and 100 ms. More details of the experimental parameters are given in the Figure captions. The data was processed with Topspin 3.6.2 (Bruker) and analyzed with Sparky 1.470 (Lee et al. 2015).

### Sample preparation for HPLC–MS/MS analysis

Ultrapure water was produced with a MilliQ® Integral 3 instrument (Millipore, Billerica, MA, USA). Triethylammonium bicarbonate buffer (TEAB, pH 8.50 ± 0.10, 1 mol L^−1^), sodium dodecyl sulfate (SDS, ≥ 99.5%), tris(2-carboxyethyl)phosphin-hydrochlorid (TCEP, ≥ 98.0%), iodoacetamide (IAA, ≥ 99.0%), formic acid (FA, 98.0–100%) and trifluoroacetic acid (TFA, ≥ 99.0%) were acquired from Sigma-Aldrich (Vienna, Austria). Methanol (MeOH, LiChrosolv®) and ortho-phosphoric acid (85%) were purchased from Merck (Darmstadt, Germany). Acetonitrile (ACN, LC–MS grade) was purchased from “VWR International” (Vienna, Austria). Trypsin (sequencing grade modified, porcine) was acquired from Promega (Madison, WI, USA). 74 µg of glycated BSA (after buffer exchange against ddH_2_O, pH 7.4, conc.: 7.4 mg^.^mL^−1^) were diluted to a concentration of 1.6 µg^.^µL^−1^ in 50 mmol L^−1^ TEAB (pH 8.50) buffer containing 5% (*w/w*) SDS and denatured by heating for 5 min at 95 °C. Next, disulfides were reduced by addition of TCEP to a concentration of 5.0 mmol L^−1^ and incubation at 55 °C for 15 min, followed by alkylation of the cysteine residues by addition of IAA to a concentration of 40 mmol L^−1^ and incubation at 22 °C in the dark for 10 min. Following, the protein was precipitated at a pH ≤ 1 with 12% (*v/v*) ortho-phosphoric acid and by adding 7:1 (*v/v*) of 100 mmol L^−1^ TEAB (pH 7.55) in 90% MeOH (*v/v*). Next, the proteins were purified by suspension trapping employing S-Trap mini columns (Protifi, Huntington, NY, USA) according to the manufacturer’s instructions, and digested to peptides employing trypsin at a protease/protein ratio of 1:10 (*w*/*w*) at 37 °C for 12 h. The obtained peptides were dried at 50 °C in a vacuum centrifuge and resuspended in 1% ACN + 0.10% FA to a concentration of 3.3 µg µL^−1^.

### High-performance liquid chromatography coupled to MS/MS

Chromatographic separation of 1.0 µg peptides was carried out in five technical replicates employing reversed-phase HPLC on an UltiMate™ 3000 RSLCnano System (Thermo Fisher Scientific, Germering, Germany), on a DNV PepMap™ Neo column (150 × 0.075 mm i.d.) from Thermo Fisher Scientific, Germering, Germany. The mobile phases used for the separation were 0.10% aqueous FA (solvent A) and 0.10% FA in ACN (solvent B), pumped at a flow rate of 200 nL^.^min^−1^ in the following order: 1.0% B for 5.0 min, a linear gradient from 1.0 to 5.0% B in 5 min, a second linear gradient from 5.0 to 35.0% B in 60.0 min, and a third linear gradient from 35.0 to 45.0% B in 20.0 min. This was followed by flushing at 99.0% B for 10 min and column re-equilibration at 1.0% B for 35 min. The column temperature for the separation was kept constant at 50 °C. The nanoHPLC system was hyphenated to a Q Exactive™ Hybrid Quadrupole-Orbitrap™ mass spectrometer via a Nanospray Flex™ ion source (both from Thermo Fisher Scientific, Bremen, Germany). The source was equipped with a SilicaTip emitter with 360 µm o.d., 20 µm i.d. and a tip i.d. of 10 µm from CoAnn Technologies Inc. (Richland, WA, USA). The spray voltage was set to 1.5 kV, S-lens RF level to 60.0 and capillary temperature to 250 °C. Each scan cycle consisted of a full scan at a scan range of *m/z* 350–2000 and a resolution setting of 70,000 at m/z 200, followed by 5 data-dependent higher-energy collisional dissociation (HCD) scans in a 2.0 m*/z* isolation window at 28% normalized collision energy at a resolution setting of 17,500 at *m/z* 200. For the full scan, the automatic gain control (AGC) target was set to 3e6 charges with a maximum injection time of 100 ms, for the HCD scans the AGC target was 1e5 charges with a maximum injection time of 150 ms. Already fragmented precursor ions were excluded for 10 s. Data acquisition was conducted using Thermo Scientific™ Chromeleon™ 7.2 CDS (Thermo Fisher Scientific, Germering, Germany). For the identification of modification sites, as well as for sequence coverage mapping, Byonic 3.11.3 (Protein Metrics, Cupertino, CA, USA) was used with a precursor and a fragment mass tolerance of 10 ppm, applying a 1% false discovery rate. Relative quantification of the modified peptides was done using MaxQuant 2.0.1.0 (Cox and Mann 2008) with the setting Label-free quantification, applying a 1% false discovery rate.

## Results

### Assignment of NMR correlation patterns of Amadori products of maltose in the model protein BSA

To obtain a suitable sample for studying the NMR correlation patterns of maltose-glycation products, we incubated bovine serum albumin (BSA) with a high concentration of maltose at pH 7.4. We chose BSA, because it produced earlier high amounts of glycation products with glucose (Moises et al. 2022). BSA contains several lysines with low pK_a_ values, which are susceptible to glycation. Specifically, we achieved glycation by incubating a solution of 11 mM BSA with 185 mM maltose for 11 days at 40 °C at pH 7.4. After buffer exchange to ddH_2_O, the treated protein was analyzed under denaturing conditions in a completely deuterated solution (7 M urea-d_4_ in D_2_O, 67 mM DTT-d_10_). Although not relevant for this study, we observed phase separation after buffer exchange of the glycated protein to ddH_2_O. However, after lyophilization, the sample completely dissolved under denaturing conditions.

**Fig. 3:**
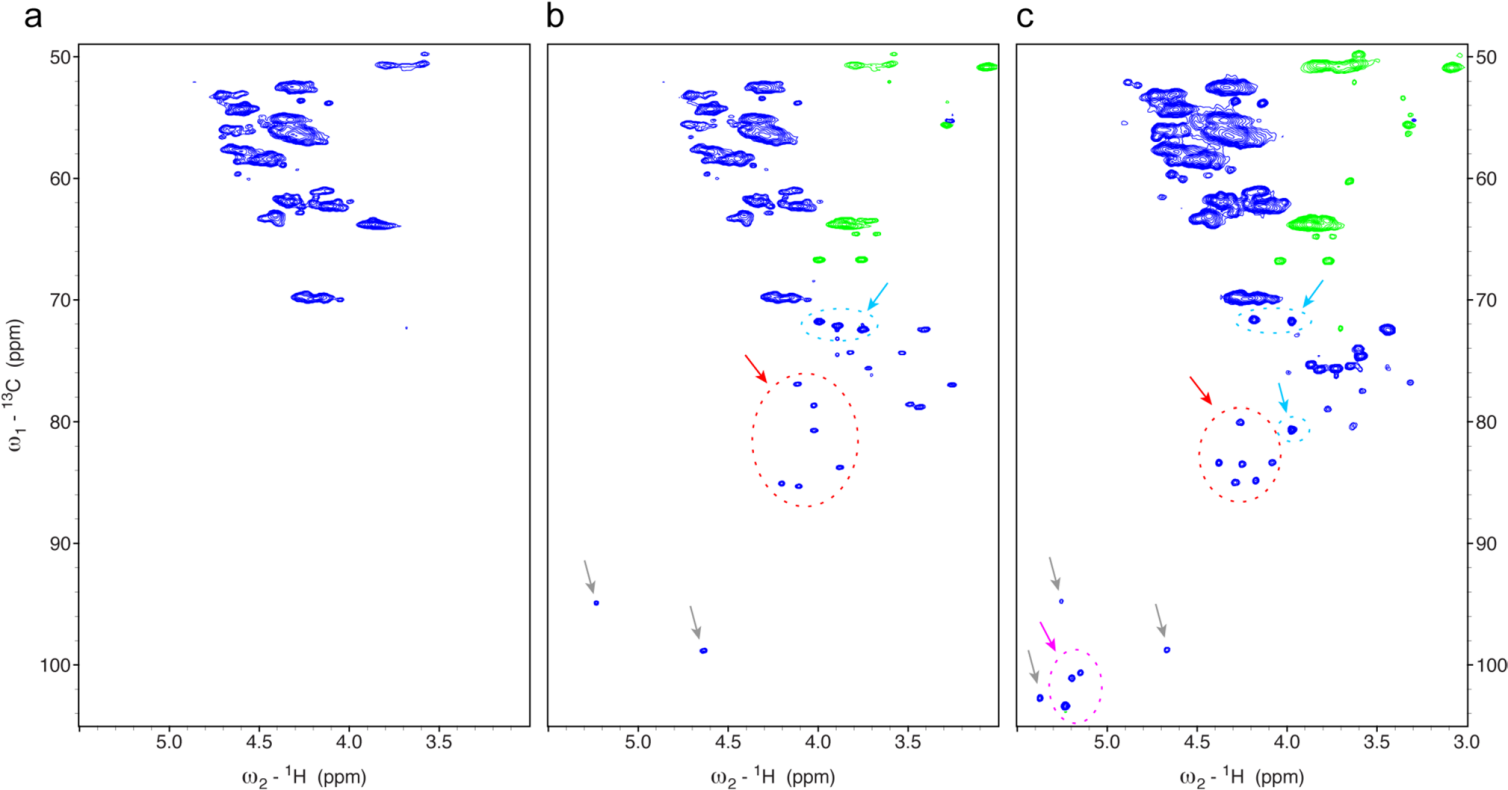
2D ^1^H-^13^C correlations comparing untreated BSA with glucose-based glycated BSA and maltose-based glycated BSA. **a** ^1^H-^13^C HSQC spectrum of untreated BSA. **b** ^1^H-^13^C HSQC spectrum of glucose-based glycated BSA. Grey arrows indicate prominent signals of free glucose. The region indicated by the red arrow is typical for the furanose forms of the Amadori product, the region marked by the cyan arrow indicates the β-pyranose form of the Amadori product. **c** ^1^H-^13^C HSQC spectrum of maltose-based glycated BSA. The same color code as in section b is used for the arrows. Interestingly, three additional anomeric signals of the Amadori product are visible indicated by a magenta arrow

The signal-to-noise was sufficient to obtain high-quality 2D NMR data for all species including less sensitive ^1^H-^13^C correlation spectra. Figure 3 shows 2D ^1^H-^13^C HSQC spectra comparing untreated BSA, glucose-based glycated BSA and maltose-based glycated BSA. Both glycated forms showed many new signals in the carbohydrate region between 71 and 105 ppm. The new sets of signals are very different for glucose-based glycated BSA compared to maltose-based glycated BSA. The anomeric region of the 2D ^1^H-^13^C HSQC spectrum (90–105 ppm) showed only in the case of maltose-based glycation three C1-H1 correlations of the terminal Glc moieties of the glycated forms in addition to signals of free sugar.

**Fig. 4:**
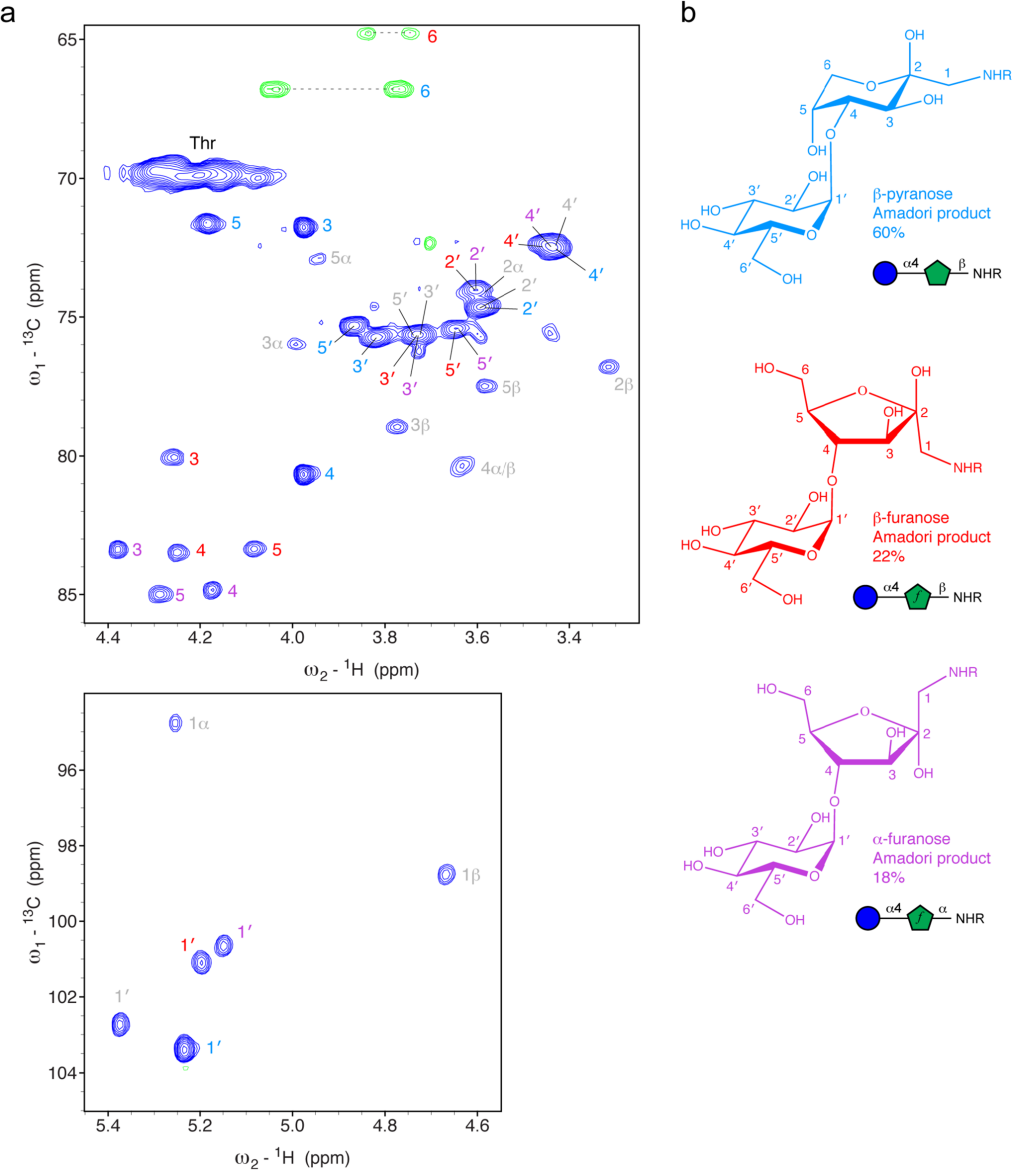
Maltose-based glycation products observed with the model protein BSA. **a** ^1^H-.^13^C HSQC spectrum of glycated BSA at a concentration of 2 mM. The sample was dialyzed against ddH_2_O, lyophilized and dissolved in 7 M urea-d4 in D_2_O pH* 7.4. The β-pyranose form, the most dominant form of the Amadori product, is labeled in cyan. The furanose forms are labeled in red and purple. The chemical structures of these species are shown on the right using the same color code. Signals of free maltose, which can form in a back-reaction, are shown with grey labels. **b** Chemical structures of the observed Amadori products with their observed abundances and symbol presentation (Varki et al. 2015; Neelamegham et al. 2019)

A detailed 2D ^1^H-^13^C HSQC spectrum of maltose-based glycated BSA with all relevant signals labeled is shown in Fig. 4. Three anomeric signals of the free maltose are visible (Glc1′, Glc1α and Glc1β) likely due to the back reaction of the reversible glycation reaction. Besides signals of free maltose in the sample, we observed three forms of Amadori products: the dominating β-pyranose form and two furanose forms with populations of 60%, 22% and 18% (Fig. 4a, b), respectively. The populations were estimated from peak volumes of the C1-H1 correlations in the 2D ^1^H-^13^C HSQC spectrum. Further, a comparison of the peak volumes with the Cβ-Hβ signal of all isoleucines (BSA contains 14 Ile residues) we could estimate an averaged glycation of 3.7 sites per BSA molecule.

**Fig. 5:**
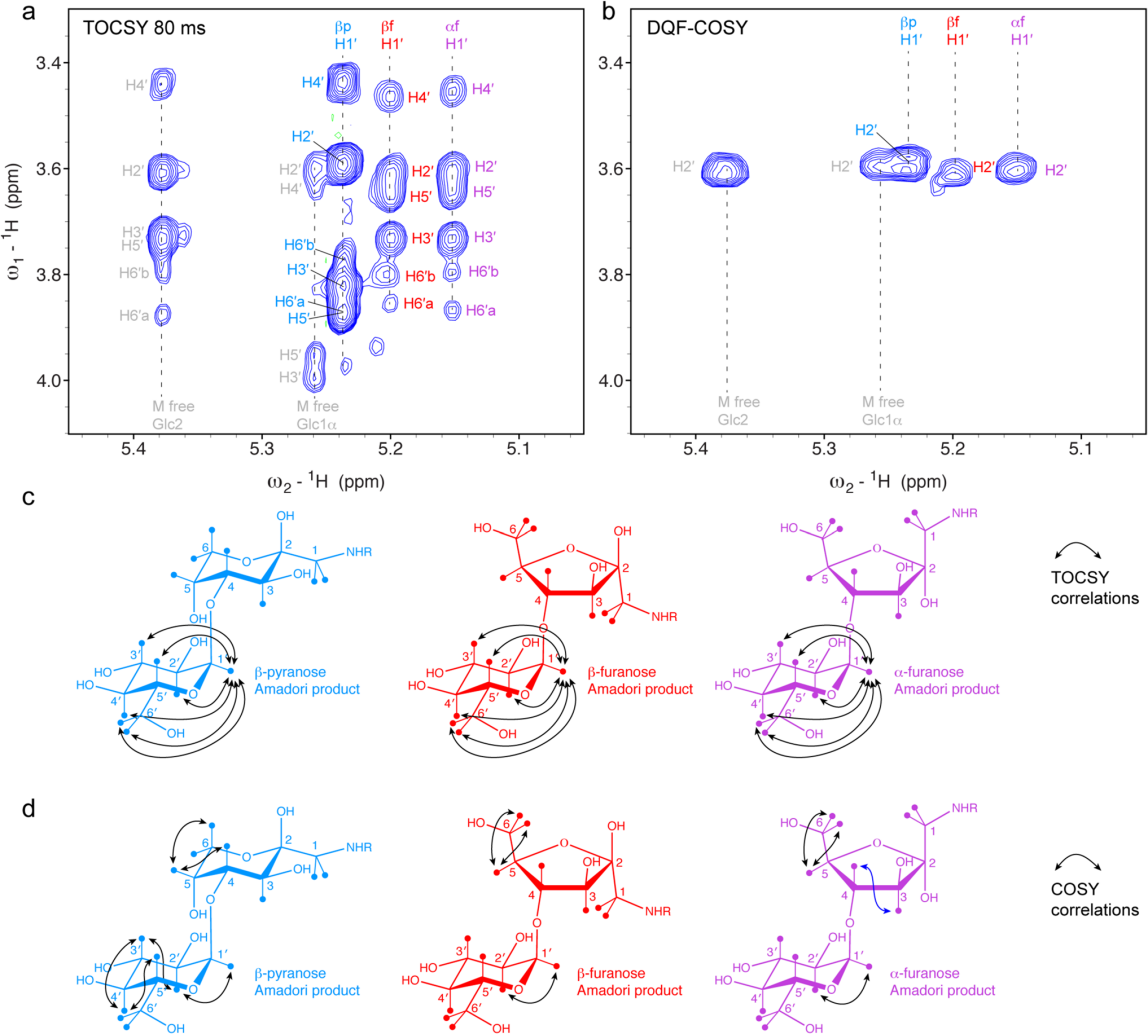
Chemical shift assignment of maltose-based glycation products observed with the model protein BSA. **a** 2D ^1^H-^1^H TOCSY spectrum with correlations of the anomeric H1 protons of the distal Glc residue of the Amadori products (cyan, red, purple) and a rest of free maltose (grey). **b** Comparable region of a 2D ^1^H-^1^H COSY spectrum with correlations of the anomeric H1 protons of the distal Glc residue of the Amadori products (cyan, red, purple) and a rest of free maltose (grey). **c** Schematic presentation of the shown TOCSY correlations on the chemical structures of all three Amadori products indicated as arrows. **d** Observed COSY correlations schematically illustrated on the chemical structures

The major form, the β-pyranose, could be readily assigned using ^1^H-^1^H TOCSY, ^1^H-^1^H COSY, ^1^H-^13^C HSQC and ^1^H-^13^C HMBC spectra (Figs. 4, 5 and Suppl. Fig. S1). The chemical shift assignment of the β-pyranose agrees with the previously reported data by (Krause et al. 2003), but the chemical shift assignments for the distal Glc moiety disagree (Table 1). However, with ^1^H-^1^H COSY, ^1^H-^1^H TOCSY and ^1^H-^13^C HSQC correlations we could unambiguously assign the distal Glc moiety (see Figs. 4 and 5). HMBC correlations between H1′ and C4 and between H4 and C1′ confirmed the linkage between the terminal Glc moiety and the first fructolysine moiety (Fig. S1).

**Table 1:** Experimental chemical shift assignment of all observed forms of maltose-based glycation products in BSA dissolved in 7 M urea-d_4_ in D_2_O (pH* 7.4) referenced to DSS at 298 K

Moiety	Atom	Observed glycated BSA	Krause 2003^a^	Observed glycated BSA	Observed glycated BSA
β-pyranose				β-furanose	α-furanose
Fructoseamine	H1/H1′	n.d	3.31	n.d	n.d
	H3	3.976	3.94	4.379	4.263
	H4	3.976	3.98	4.174	4.257
	H5	4.184	4.19	4.289	4.084
	H6	4.045	4.03	3.868	3.844
	H6′	3.770	3.74	3.767	3.749
	C1	n.d	55.5	n.d	n.d
	C2	n.d	98.3	n.d	n.d
	C3	71.8	71.8	83.37	80.04
	C4	80.7	80.4	84.84	83.49
	C5	71.6	71.7	84.99	83.36
	C6	66.8	66.8	63.80	64.80
Distal Glc	H1	5.234	5.24	5.149	5.201
	H2	3.59	3.84^b^	3.606	3.611
	H3	3.823	3.43^b^	3.732	3.731
	H4	3.437	3.80^b^	3.452	3.461
	H5	3.873	3.59^b^	3.643	3.649
	H6	3.877	3.82^b^	3.866	3.857
	H6′	3.777	3.82^b^	3.799	3.795
	C1	103.4	103.4	100.70	191.10
	C2	74.6	75.2^b^	74.01	74.00
	C3	75.7	72.4^b^	75.62	75.61
	C4	72.5	75.6^b^	72.42	72.42
	C5	75.3	74.5^b^	75.40	75.43
	C6	63.8	63.3	63.32	63.37
Glycated lysine	HE	3.086	3.09	n.d	n.d
	HD	1.747	1.74	n.d	n.d
	HG	1.45	1.42	n.d	n.d
	CE	50.9	51.1	n.d	n.d
	CD	27.6	27.5	n.d	n.d
	CG	25.3	24.9	n.d	n.d

^a^The reported chemical shifts had an offset, for better comparison all reported ^13^C chemical shifts were corrected here by adding 3.2 ppm, all reported ^1^H chemical shifts by adding 0.14 ppm

^b^The assignment seems to be mixed up, their C2 seems to be our C5, their C3 our C4, their C4 our C3, their C5 our C2; for ^1^H it seems to be more difficult

**Fig. 6:**
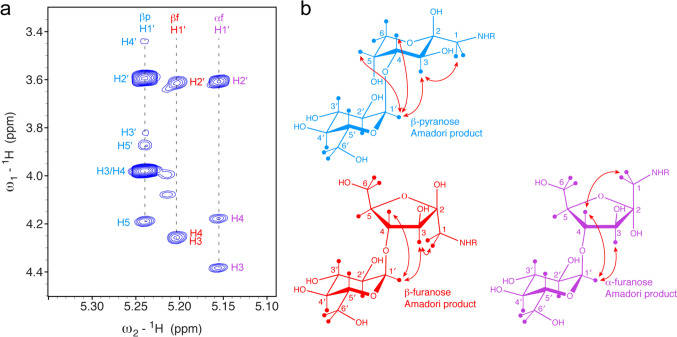
Key NOE correlations of maltose-based glycation products observed with the model protein BSA. **a** 2D ^1^H-^1^H NOESY spectrum of the anomeric H1 protons of the distal Glc residue showing correlations to the proximal fucose residues for all three observed Amadori products. **b** Observed NOE correlations schematically shown on the chemical structures

For the assignment of the minor forms, we recorded in addition ^1^H-^1^H NOESY (Fig. 6) and ^1^H-^13^C HSQC-TOCSY spectra with different mixing times (data not shown). The ^1^H-^13^C HSQC-TOCSY spectrum with a short mixing time (13 ms) shows typically at one ^13^C frequency correlations to three ^1^H frequencies—of the directly attached ^1^H and of the next ^1^H neighbors. Almost complete chemical shift assignments were obtained, and the three sets of products could be unambiguously assigned to the β-pyranose, β-furanose and α-furanose forms. In addition, the random coil chemical shifts of the modified lysines could be assigned (Table 1). Here especially the Cε–Hε correlation is characteristic. It is very similar to glucose-based glycation.

### Independent confirmation of maltose-based glycation by MS/MS analysis

**Fig. 7:**
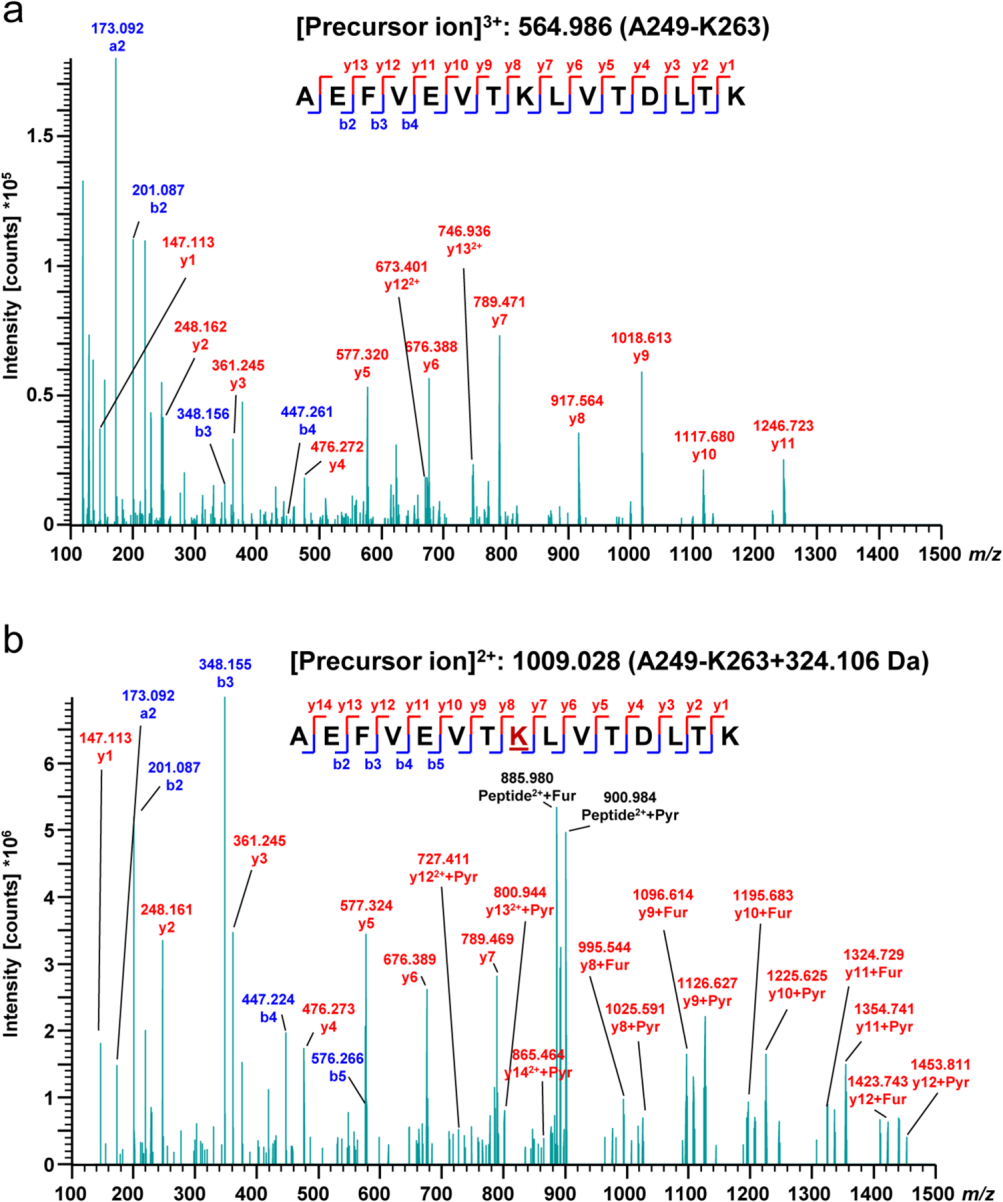
Exemplary MS/MS spectra of the BSA peptide (Ala249 –Lys263) unmodified [uncharged mass 1691.935 Da] (**a**) and glycated at Lys256 [uncharged mass 2016.040 Da] (**b**), corresponding to a delta mass of 324.106 Da. The sequence of the peptide is almost completely covered by y ions in both cases. In the maltosylated variant (**b**), the fragments from y8-y14 are observed as furylium (+ 78 Da) and pyrilium (+ 108 Da), representing typical dissociation pathways for peptides glycated with aldohexoses

BSA glycated by maltose was independently analyzed by HPLC-MS/MS analysis. Figure 7 shows exemplary data of the peptide (Ala249 –Lys263) unmodified and glycated at Lys256. A mass difference of 324.106 Da was observed for the parent ions corresponding to a modification of maltose consisting of two hexoses. The sequence of the peptide is almost completely covered by y ions in both cases. In the maltosylated variant (Fig. 7b), the fragments from y8 to y14 are observed as furylium (+ 78 Da) and pyrylium (+ 108 Da), products of typical dissociation pathways for peptides glycated with aldohexoses (Corzo-Martinez et al. 2009). The sequence of BSA was well covered (Supplementary Fig. S2) providing evidence for the glycation of 51 out of all 59 lysine residues (Supplementary Fig. S3 and Table S1). The individual abundances of glycation ranged from 0.4 to 98.2% (Suppl. Table S1).

### Detection of maltose-based glycation in a biotherapeutic Fc-fusion protein

In contrast to glycation with Glc, whose Amadori product lacked an anomeric proton, the anomeric signals of the terminal Glc moiety of the maltose-Amadori product are well separated for the different forms in a ^1^H-^1^H TOCSY (Fig. 5). More importantly, they occur in a characteristic region, which is normally quite empty and not disturbed by neither protein nor glycosylation signals. These patterns of chemical shift correlations can be used to unambiguously identify the presence of maltose-based glycation in any protein sample.

**Fig. 8:**
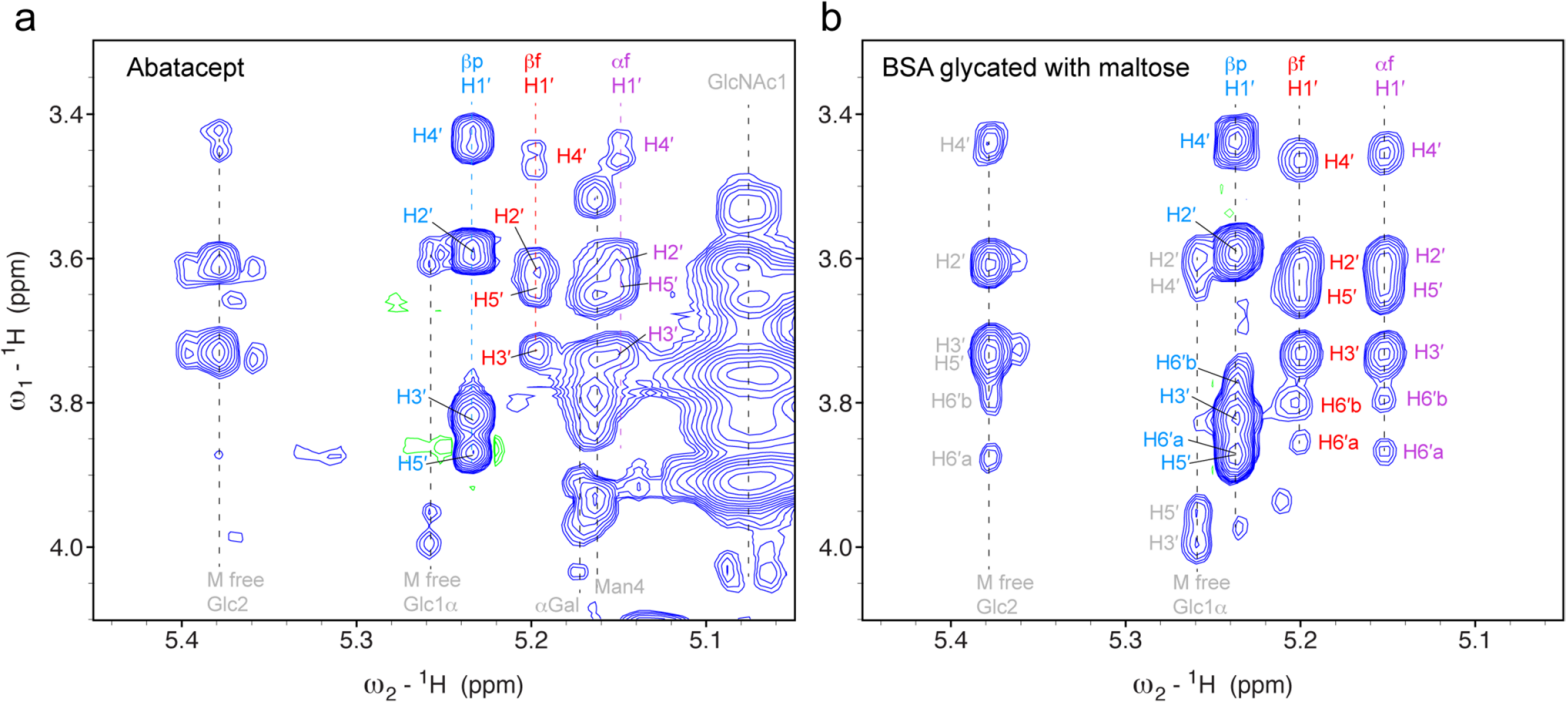
Unambiguous identification of maltose-based glycation in the biotherapeutic abatacept. **a** 2D ^1^H-^1^H TOCSY spectrum of the dialyzed and lyophilized abatacept dissolved in 7 M urea-d_4_ in D_2_O (pH* 7.4) showing the anomeric region. Correlations of all three forms of maltose-based glycation are visible despite the busy background of n-glycosylation signals. **b** Comparable region of a 2D ^1^H-^1^H TOCSY spectrum of maltose-glycated BSA

We decided to analyze the biotherapeutic abatacept, which is stored with a high amount of maltose in its formulation buffer and of which maltose-glycation was reported earlier (Lynaugh et al. 2013). Figure 8a shows the relevant region of a 2D ^1^H-^1^H TOCSY of abatacept under denaturing conditions in comparison to the here assigned maltose-based glycated BSA (Fig. 8b).

The presence of maltose-based glycation in a protein is indicated by a characteristic pattern of 4 strong signals of the β-pyranose form. Both fucose forms give additionally very characteristic patterns consisting of 6 signals each, however, with lower intensity due to their lower abundance. Monitoring this region of a 2D ^1^H-^1^H TOCSY is an ideal method to unambiguously recognize maltose adducts in proteins, e.g. in biotherapeutic proteins or proteins within foods. ^1^H-^13^C HSQC correlations are also very characteristic and unique. However, such ^1^H-^13^C spectra are less sensitive due to the low natural abundance of ^13^C of only 1.1% and require therefore much longer measurement times like 24 h to obtain a sufficient signal-to-noise ratio. From comparing the weak C4–H4 signal of the proximal ring of the β-pyranose form in the ^1^H-^13^C HSQC spectrum and Cβ-Hβ signal of all Ile residues (abatacept dimer contains 22 Ile residues), we estimate that on average 16.7% of all Fc-fusion dimers are glycated.

## Discussion

The chemical shift patterns of the maltose-based glycation products in 2D NMR spectra are clearly different than those of glucose-based glycation. This allows the unambiguous distinction between these two different modifications due to glycation resulting from different sugars with free reducing ends.

The characteristic signals of the terminal Glc of maltose, which are well isolated in a sensitive ^1^H-^1^H TOCSY spectrum, enable the straightforward and sensitive detection of maltose glycation. This stands in contrast to glycation by the monosaccharide glucose whose anomeric signal is lost after the Amadori rearrangement, and whose presence is only detectable by characteristic NMR correlation patterns using ^1^H-^13^C HSQC correlations, which are much less sensitive due to the low natural abundance of ^13^C of 1.1%. Here ^1^H-^1^H TOCSY spectra can be recorded within the time of approx. 1 h depending on the recycle delay and the resolution chosen in the indirect dimension. It is not so common to have isolated and characteristic signals of PTMs in ^1^H-^1^H TOCSY spectra, because dispersion in ^1^H is much smaller than in ^13^C, but rare cases like characteristic signals of α-Gal epitopes (Hinterholzer et al. 2022) and now maltose-glycation allow the unambiguous detection in a time efficient and easy to interpret manner.

The observed populations of the different Amadori products were different compared to glucose-based glycation. We observed here 60% β-pyranose, 22% β-furanose and 18% α-furanose for maltose-based glycation, whereas the α-pyranose was either absent or too weak to be observed. For glucose-based glycation the populations were 70% β-pyranose, 13% β-furanose, 13% α-furanose and 4% α-pyranose (Mossine et al. 1994; Moises et al. 2022). This indicates that the α1,4-linked glucose substitution influences the equilibrium of the Amadori products: although the β-pyranose form is still dominating, it is less populated; both furanose forms are more abundant, but the β-furanose is favored.

The lower detection limit of the approach is in principle independent of the kind of modification. We determined in an earlier study an absolute amount of ~ 28 nmol for the oxidation product of methionine using ^1^H-^13^C HSQC spectra recorded in 24 h on a 900 MHz spectrometer with cryogenic probe (Hinterholzer et al. 2020). However, this depends on the sensitivity of the spectrometer and the measurement time. A similar detection limit was reported by Peng et al. (Peng et al. 2018). In the case of the denatured abatacept sample with a concentration of 1.2 mM (dimer), this lower limit corresponds to an average modification of 5% of all dimers. For ^1^H-^1^H correlations it will be even lower.

The strength of the presented NMR approach is the unambiguous identification of maltose-based glycation and the possibility of quantifying the total amount. A weakness is that it does not provide sequence specific localization and that the detection limit of NMR is orders of magnitudes higher than MS-based techniques. However, it is quite complementary to HPLC-MS^2^ methods, which are much more sensitive and can provide sequence specific modifications, but quantification is less accurate and identification of a modification is sometimes ambiguous. The same mass difference can have several origins, e.g. a glucose-based glycation site or an extra hexose within a glycan, or a maltose-based glycation within a peptide versus two glucose-based glycation sites. Another disadvantage of HPLC-MS^2^ methods is that the investigated modification should typically be known beforehand to set up the experiments appropriately. Another advantage of NMR spectroscopy is that with either a single spectrum (HSQC) or two spectra (TOCSY in addition) one gets an overview of many different modifications that are present in a sample, e.g. a therapeutic protein. Even unknown modifications could be detected if they result in characteristic patterns. The complementarity of the methods allows the unambiguous identification of a certain type of modification with NMR, to detect it with much lower sensitivity with MS-based techniques and to cross-validate or even calibrate MS-based quantifications.

It did not escape our attention that the maltose-glycation of a protein might serve as a very simple model system to study detailed mechanisms of reductive amination. The proximal glucose at the reducing end might serve as a model for any glucose at the reducing end and the distal glucose is a very simple substitution so that the system can still be easily studied. That is much different compared to studying larger oligosaccharides or long polysaccharides linked to proteins by reductive amination, particles or surfaces (Gildersleeve et al. 2008; Munster et al. 2017). In the case of a polysaccharide-protein conjugate the abundance and signal intensity of the reducing end is typically very low and NMR line widths are much larger at the connection point of two polymers making it practically impossible to observe small populations of reaction intermediates or the products at the linkage site. Functionalized particles or surfaces are even more difficult to study.

In conclusion, we found characteristic NMR correlation patterns of maltose-based glycation in ^1^H-^13^C HSQC and in ^1^H-^1^H TOCSY spectra that are suitable to unambiguously identify the presence of glycation by maltose. The detection by very sensitive ^1^H-^1^H TOCSY spectra is very competitive, because it relies exclusively on ^1^H nuclei with 99.99% abundance in contrast to ^13^C with only 1.11%. This approach is complementary to MS-based methods, it is suited as an independent standard for cross-validation.

### Supplementary Information

Below is the link to the electronic supplementary material.

Supplementary file1 (PDF 505 KB)
