# Supplementary material for: The NMR signature of maltose-based glycation in full-length proteins
Source: J Biomol NMR. 2023 Dec 20;78(1):61–72. doi: 10.1007/s10858-023-00432-5 (PMC10981599; doi:10.1007/s10858-023-00432-5)
Supplement: Supplementary file 1 — Supplementary file1 (PDF 505 KB) [file 10858_2023_432_MOESM1_ESM.pdf]

# Supplementary Material

## The NMR signature of maltose-based glycation in full-length proteins

Pauline Defant[1], Christof Regl[1], Christian G. Huber[1] and Mario Schubert[1,2*]

[1] Department of Biosciences and Medical Biology, University of Salzburg, Hellbrunnerstrasse 34, 5020 Salzburg, Austria.

[2] Department of Biology, Chemistry and Pharmacy, Free University of Berlin, Takustr. 3, 14195 Berlin, Germany.

## Supplementary Figures

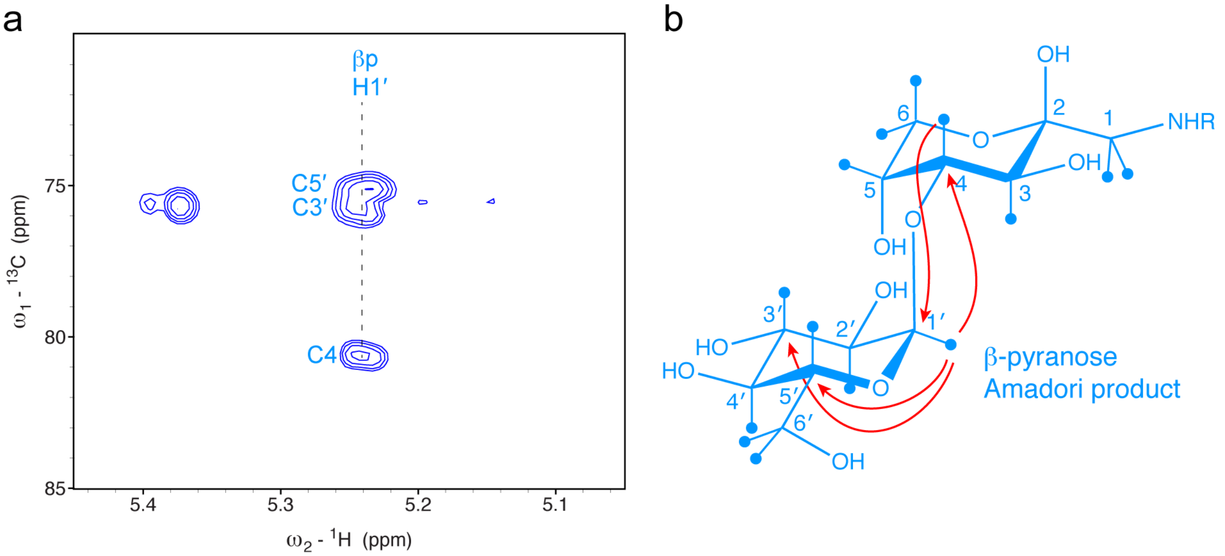


**Supplementary Figure S1. Observed $^{1}$H-$^{13}$C long-range correlations of the major form of the glycation product of BSA with maltose.** a) Part of a 2D $^{1}$H-$^{13}$C HMBC spectrum showing correlations to the anomeric H1 of the distal glucose of the major β-pyranose form.

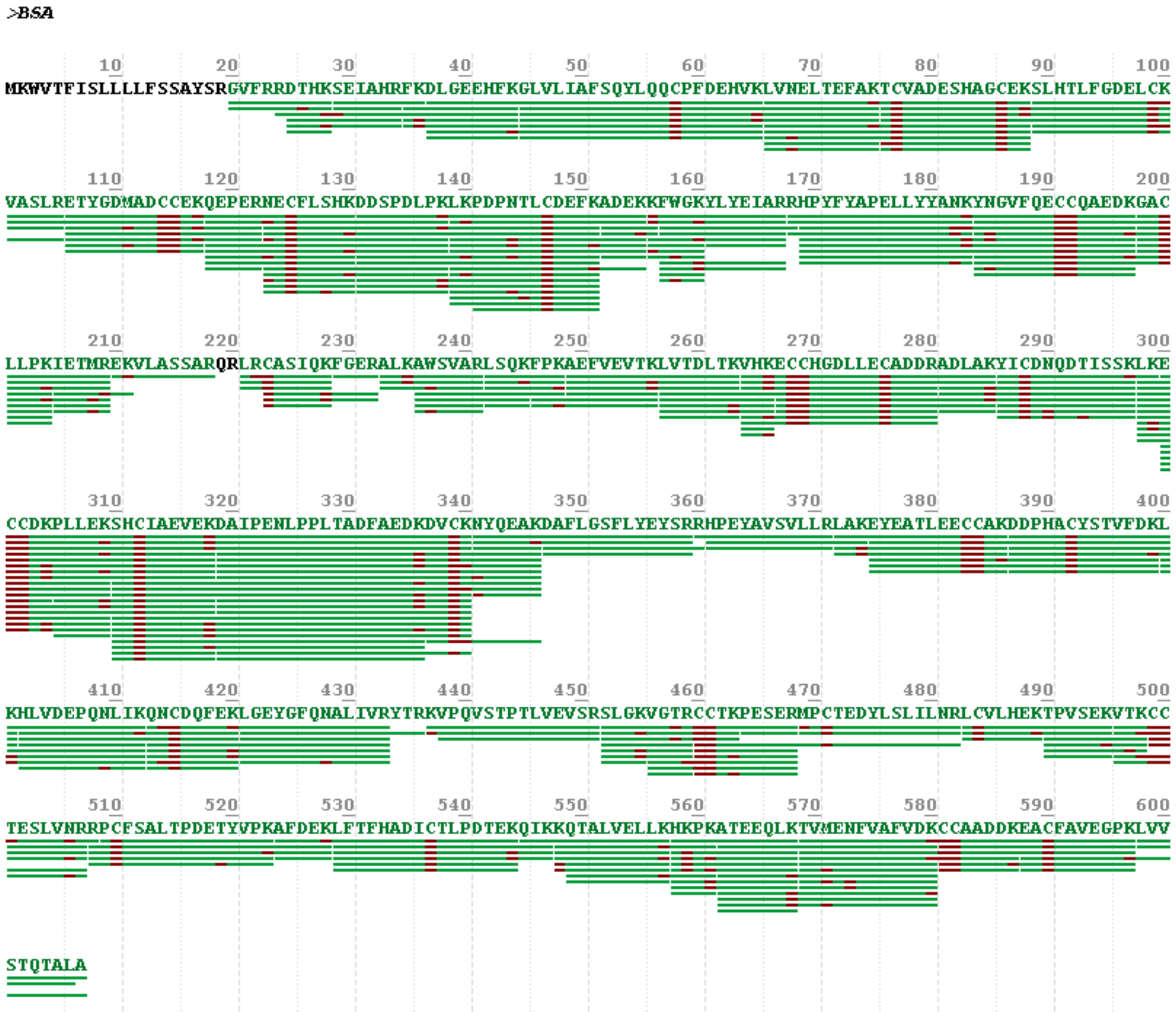


**Supplementary Figure S2. Coverage of the BSA sequence by the MS/MS analysis**. Identified peptides are indicated in green, modified amino acids are shown in red (e.g. with glycated Lys, alkylated Cys, deamidation, phosphorylation or oxidation).

```
        10         20         30         40         50
         |          |          |          |          |
MKWVTFISLL LLFSSAYSRG VFRRDTHKSE IAHRFKDLGE EHFKGLVLIA
        60         70         80         90        100
         |          |          |          |          |
FSQYLQQCPF DEHVKLVNEL TEFAKTCVAD ESHAGCEKSL HTLFGDELCK
       110        120        130        140        150
         |          |          |          |          |
VASLRETYGD MADCCEKQEP ERNECFLSHK DDSPDLPKLK PDPNTLCDEF
       160        170        180        190        200
         |          |          |          |          |
KADEKKFWGK YLYEIARRHP YFYAPELLYY ANKYNGVFQE CCQAEDKGAC
       210        220        230        240        250
         |          |          |          |          |
LLPKIETMRE KVLASSARQR LRCASIQKFG ERALKAWSVA RLSQKFPKAE
       260        270        280        290        300
         |          |          |          |          |
FVEVTKLVTD LTKVHKECCH GDLLECADDR ADLAKYICDN QDTISSKLKE
       310        320        330        340        350
         |          |          |          |          |
CCDKPLLEKS HCIAEVEKDA IPENLPPLTA DFAEDKDVCK NYQEAKDAFL
       360        370        380        390        400
         |          |          |          |          |
GSFLYEYSRR HPEYAVSVLL RLAKEYEATL EECCAKDDPH ACYSTVFDKL
       410        420        430        440        450
         |          |          |          |          |
KHLVDEPQNL IKQNCDQFEK LGEYGFQNAL IVRYTRKVPQ VSTPTLVEVS
       460        470        480        490        500
         |          |          |          |          |
RSLGKVGTRC CTKPESERMP CTEDYLSLIL NRLCVLHEKT PVSEKVTKCC
       510        520        530        540        550
         |          |          |          |          |
TESLVNRRPC FSALTPDETY VPKAFDEKLF TFHADICTLP DTEKQIKKQT
       560        570        580        590        600
         |          |          |          |          |
ALVELLKHKP KATEEQLKTV MENFVAFVDK CCAADDKEAC FAVEGPKLVV

STQTALA
```

**Supplementary Figure S3. Graphical representation of the BSA (Uniprot ID P02769) sequence coverage.** The areas in green were identified in one or more peptides, with a total sequence coverage of 96.5%, black areas were not identified. Red underlined lysine residues were identified with a +324 Da modification corresponding to glycation with maltose.

## Supplementary Tables

**Table S1** Observed peptide fragments in the MS/MS analysis.

| Peptides containing Lysine residues | Residue | Maltosylation [%] ± C.I. 95% (n=5) |
|---|---|---|
| DTHKSEIAHR | K(28) | 78.4 ± 5.0 |
| FKDLGEEHFK | K(36) | 3.2 ± 0.4 |
| DLGEEHFKGLVLIAFSQYLQQCPFDEHVK | K(44) | 0.7 ± 0.6 |
| GLVLIAFSQYLQQCPFDEHVKLVNELTEFAK | K(65) | 63.8 ± 26.2 |
| LVNELTEFAKTCVADESHAGCEK | K(75) | 77.0 ± 2.4 |
| TCVADESHAGCEKSLHTLFGDELCK | K(88) | 75.6 ± 0.8 |
| SLHTLFGDELCKVASLR | K(100) | 35.6 ± 4.0 |
| ETYGDMADCCEKQEPER | K(117) | 80.3 ± 2.8 |
| NECFLSHKDDSPDLPK | K(130) | 1.4 ± 0.6 |
| DDSPDLPKLKPDPNTLCDEFK | K(138) | 3.7 ± 1.2 |
| LKPDPNTLCDEFK | K(140) | 3.4 ± 2.9 |
| PDPNTLCDEFK | K(151) | 0 ± 0 |
| ADEKKFWGK | K(155) | 0 ± 0 |
| KFWGKYLYEIAR | K(156) | 98.2 ± 0.5 |
| FWGKYLYEIAR | K(160) | 89.6 ± 0.5 |
| HPYFYAPELLYYANKYNGVFQECCQAEDK | K(183) | 57.3 ± 9.6 |
| YNGVFQECCQAEDKGACLLPK | K(197) | 56.1 ± 5.2 |
| GACLLPKIETMR | K(204) | 58.6 ± 6.2 |
| GACLLPKIETMREK | K(211) | 0 ± 0 |
| LRCASIQK | K(228) | 0 ± 0 |
| ALKAWSVAR | K(235) | 93.2 ± 3.7 |
| AWSVARLSQK | K(245) | 0 ± 0 |
| FPKAEFVEVTK | K(248) | 73.9 ± 2.7 |
| AEFVEVTKLVTDLTK | K(256) | 75.8 ± 4.9 |
| LVTDLTKVHK | K(263) | 32.8 ± 7.2 |
| VHK(1)ECCHGDLLECADDR | K(266) | 74.5 ± 0.7 |
| ADLAKYICDNQDTISSK | K(285) | 86.7 ± 2.5 |
| YICDNQDTISSKLK | K(297) | 47.1 ± 4.5 |
| LKECCDKPLLEK | K(299) | 9.5 ± 3.4 |
| ECCDKPLLEK | K(304) | 3.1 ± 1.3 |
| PLLEKSHCIAEVEK | K(309) | 5.3 ± 2.6 |
| SHCIAEVEKDAIPENLPPLTADFAEDKDVCK | K(318) | 1.4 ± 0.5 |
| SHCIAEVEKDAIPENLPPLTADFAEDKDVCK | K(336) | 0.4 ± 0.4 |
| DVCKNYQEAK | K(340) | 1.5 ± 0.9 |

| | | |
|---|---|---|
| NYQEAKDAFLGSFLYEYSR | K(346) | 86.7 ± 21.7 |
| LAKEYEATLEECCAK | K(374) | 82.9 ± 1.8 |
| EYEATLEECCAKDDPHACYSTVFDK | K(386) | 47.2 ± 4.1 |
| DDPHACYSTVFDKLK | K(399) | 87.9 ± 1.8 |
| LKHLVDEPQNLIK | K(401) | 80.1 ± 5.5 |
| HLVDEPQNLIKQNCDQFEK | K(412) | 81.8 ± 5.3 |
| QNCDQFEKLGEYGFQNALIVR | K(420) | 89.8 ± 1.9 |
| KVPQVSTPTLVEVSR | K(437) | 1.8 ± 0.2 |
| SLGKVGTR | K(455) | 83.5 ± 1.1 |
| CCTKPESER | K(463) | 4.5 ± 1.0 |
| LCVLHEKTPVSEK | K(489) | 80.7 ± 3.6 |
| TPVSEKVTKCCTESLVNR | K(495) | 62.4 ± 41.1 |
| VTKCCTESLVNR | K(498) | 31.6 ± 6.2 |
| RPCFSALTPDETYVPKAFDEK | K(523) | 95.4 ± 1.8 |
| AFDEKLFTFHADICTLPDTEK | K(528) | 90.5 ± 1.9 |
| LFTFHADICTLPDTEKQIK | K(544) | 0 ± 0 |
| LFTFHADICTLPDTEKQIK | K(547) | 0 ± 0 |
| KQTALVELLK | K(548) | 36.5 ± 1.4 |
| QTALVELLK | K(557) | 0 ± 0 |
| HKPKATEEQLK | K(559) | 57.0 ± 7.3 |
| HKPKATEEQLK | K(561) | 63.7 ± 56.7 |
| ATEEQLKTVMENFVAFVDK | K(568) | 2.8 ± 0.7 |
| TVMENFVAFVDKCCAADDKEACFAVEGPK | K(580) | 8.9 ± 3.1 |
| CCAADDKEACFAVEGPK | K(587) | 0.4 ± 0.1 |
| EACFAVEGPKLVVSTQTALA | K(597) | 0.4 ± 0.1 |
| | Total | 41.8 ± 4.6 |
